# Supplementary material for: Biting behaviour and infectivity of Simulium damnosum complex with Onchocerca parasite in Alabameta, Osun State, Southwestern, Nigeria
Source: PLoS One. 2021 Jun 4;16(6):e0252652. doi: 10.1371/journal.pone.0252652 (PMC8177508; doi:10.1371/journal.pone.0252652)
Supplement: S1 File — (DOCX) [file pone.0252652.s001.docx]

**CASE STUDY FORM**

**Project Title**

Biting behaviour and infectivity of *Simulium damnosum* complex with Onchocerca parasite in Alabameta, Osun State, Southwestern, Nigeria

**Location**

Alabameta community, Osun State, Nigeria

**Summary**

The study is targeted at providing baseline information to assist in the global effort towards the control and eradication of Onchocerciasis in Nigeria and the world at large. A neglected tropical disease (NTD) with an alarming and worrisome burden globally. Complementing efforts of the Africa Programme for Onchocerciasis Control (APOC) and other health organizations and non-governmental organizations (NGOs).

**Process summary**

| **Year** | **Month** | **Activity** |
| --- | --- | --- |
| 2014 | November | Fly collection/dissection |
| 2014 | December | Fly collection/dissection |
| 2015 | January | Fly collection/dissection |
| 2015 | March | Fly collection/dissection |
| 2015 | April | Fly collection/dissection/data analysis |

**Process**

| **Year** | **Month** | **Activity** |  |
| --- | --- | --- | --- |
| 2014 | November | Fly collection/dissection(morphological identification/infectivity/parity) |  |
| 2014 | December | Fly collection/dissection(morphological identification/infectivity/parity) |  |
| 2015 | January | Fly collection/dissection(morphological identification/infectivity/parity) |  |
| 2015 | March | Fly collection/dissection(morphological identification/infectivity/parity) |  |
| 2015 | April | Fly collection/dissection(morphological identification/infectivity/parity)/data analysis |  |

**Context**

Among the filarial nematodes, the public health significance of *Onchocerca volvulus* cannot be overemphasized being the causative organism of the dreadful and debilitating disease onchocerciasis. Human onchocerciasis (river blindness) causes blindness and severe dermatitis in Africa and Latin America (WHO, 1976) and is the second leading cause of blindness (Dent and Kazura, 2011). Onchocerciasis is transmitted by members of *Simulium damnosum* complex through their bite while taking a blood meal (Yameogo *et al*., 1999).

The aforementioned processes were conducted to:

- To determine the species composition of black flies in the study area
- To investigate the infectivity of black flies in the study area
- To determine the population dynamics of the vector in the study area
