# Supplementary material for: Biting behaviour and infectivity of Simulium damnosum complex with Onchocerca parasite in Alabameta, Osun State, Southwestern, Nigeria
Source: PLoS One. 2021 Jun 4;16(6):e0252652. doi: 10.1371/journal.pone.0252652 (PMC8177508; doi:10.1371/journal.pone.0252652)
Supplement: Attachment — Submitted filename: Response to Reviewers.docx [file pone.0252652.s002.docx]

Department of Zoology,

Osun State University,

P.M.B 4494,

Osogbo,

Osun State,

Nigeria.

1st April, 2021.

The Editorial Manager,

PLOS One,

San Francisco,

California,

United States of America.

Sir,

**REVISED MANUSCRIPT ON BITING BEHAVIOUR AND INFECTIVITY OF *Simulium damnosum* complex** **WITH ONCHOCERCA PARASITE IN ALABAMETA, OSUN STATE, SOUTHWESTERN, NIGERIA’**

Sequel to your comments on the manuscript ‘Biting behaviour and infectivity of *Simulium damnosum* complex with Onchocerca parasite in Alabameta, Osun State, Southwestern, Nigeria’, I write to inform you that the authors have responded to the comments raised by the reviewers. Specifically, kindly find below detailed response:

1. The issue on Figure 2 has be revised. The months are now included in the y-axis, and consistency in the spacing within the figures.
2. The Ethical Clearance was obtained from UNIOSUN-HREC. It has been reflected in the manuscript and the letter is uploaded as supplementary document.
3. The statement on zero infectivity has been revised to reflect possibility of zero infectivity since our study did not deal with 6,000 flies.

It is our fervent hope that the Editor would find the revised manuscript suitable for publication.

Yours faithfully,

Signed,

Busari, Lateef Oluwatoyin.
