# Supplementary material for: Biting behaviour and infectivity of Simulium damnosum complex with Onchocerca parasite in Alabameta, Osun State, Southwestern, Nigeria
Source: PLoS One. 2021 Jun 4;16(6):e0252652. doi: 10.1371/journal.pone.0252652 (PMC8177508; doi:10.1371/journal.pone.0252652)
Supplement: Attachment — Submitted filename: .Response to Reviewers.docx [file pone.0252652.s003.docx]

Department of Zoology,

Osun State University,

P.M.B 4494,

Osogbo,

Osun State,

Nigeria.

15th May, 2021.

The Editorial Manager,

PLOS One,

San Francisco,

California,

United States of America.

Sir,

**REVISED MANUSCRIPT ON BITING BEHAVIOUR AND INFECTIVITY OF *Simulium damnosum* complex** **WITH ONCHOCERCA PARASITE IN ALABAMETA, OSUN STATE, SOUTHWESTERN, NIGERIA’**

Sequel to your comment on the manuscript ‘Biting behaviour and infectivity of *Simulium damnosum* complex with Onchocerca parasite in Alabameta, Osun State, Southwestern, Nigeria’, I write to inform you that the authors have responded to the comment raised by the reviewers on the repetition of the ethical clearance statement twice by removing the one previously on line 65-67.

It is our fervent hope that the Editor would find the revised manuscript suitable for publication.

Yours faithfully,

Signed,

Busari, Lateef Oluwatoyin.
